# Descriptive statistics and risk factor analysis of children with community-acquired septic shock

**DOI:** 10.1186/s40560-023-00652-9

**Published:** 2023-02-13

**Authors:** Shinya Miura, Nobuaki Michihata, Yohei Hashimoto, Hiroki Matsui, Kiyohide Fushimi, Hideo Yasunaga

**Affiliations:** 1 Department of Pediatrics, St. Marianna University School of Medicine, 2 Chome-16-1 Sugao, Miyamae Ward, Kawasaki, Kanagawa Japan; 2 Teikyo University Graduate School of Public Health, Tokyo, Japan; 3 Department of Health Services Research, Graduate School of Medicine, The University of Tokyo, Tokyo, Japan; 4 Department of Ophthalmology, Graduate School of Medicine, The University of Tokyo, Tokyo, Japan; 5 Department of Clinical Epidemiology and Health Economics, School of Public Health, The University of Tokyo, Tokyo, Japan; 6 Department of Health Policy and Informatics, Tokyo Medical and Dental University Graduate School, Tokyo, Japan

**Keywords:** Sepsis, Septic shock, Community-acquired, Epidemiology, Children, Pediatric

## Abstract

**Background:**

Children with community-acquired septic shock can rapidly deteriorate and die in acute-care hospitals. This study aimed to describe the mortality, timing, and risk factors in children with community-acquired septic shock.

**Methods:**

This is a retrospective cohort study using a national inpatient database in Japan. The study population included children (age < 20 years) who were admitted to acute-care hospitals with a diagnosis of sepsis from July 2010 to March 2020, who were treated with antibiotics, and who were supported with vasoactive drugs within three days of hospitalization. We used a Cox proportional-hazards regression model to identify risk factors for earlier death.

**Results:**

Among 761 eligible children, the median age was 3 (interquartile range, 0–11) years and 57.2% had underlying conditions. Among these, 67.1% were admitted to accredited intensive care units within three days of hospitalization and 38.6% were transported from other hospitals. The median hospital volume, defined as the number of eligible children in each hospital over the study period, was 4 (interquartile range, 2–11). Overall, 244 children died (in-hospital mortality rate, 32.1%). Among them, 77 (31.6%) died on the first day, and 156 (63.9%) died within three days of hospitalization. A Cox proportional-hazards regression model showed that earlier death was associated with lower hospital volume and age 1–5 years, whereas it was inversely associated with admission to an accredited intensive care unit and transport from other hospitals. Among 517 survivors, 178 (34.4%) were discharged with comorbidities.

**Conclusions:**

Children with community-acquired septic shock had high mortality, and early death was common. Our findings may warrant future efforts to enhance the quality of initial resuscitation for sepsis in low-volume hospitals and to ensure a healthcare system in which children with sepsis can be treated in accredited intensive care units.

## Introduction

Sepsis is defined as life-threatening organ dysfunction caused by a dysregulated host response to infection [1]. Sepsis accounts for 10–20% of all deaths in children [2, 3]. Among sepsis-related deaths in children, 30–50% of deaths occur within the first few days after the recognition of sepsis [4–7]. Septic shock is associated with even higher mortality and early death, suggesting the importance of initial resuscitation [8]. Community-acquired septic shock has been considered an important issue from a public health perspective due to its incidence and economic burden in comparison to hospital-acquired septic shock [9, 10].

There is accumulating evidence of community-acquired septic shock in children admitted to pediatric intensive care units (PICUs). Community-acquired septic shock accounts for around half of pediatric septic shock [10, 11]. In-hospital mortality remains around 10%, even in high-income countries [11, 12]. In addition, more than one-third of hospital survivors suffer from a reduced quality of life after hospital discharge [13].

In contrast, data on community-acquired septic shock in children admitted to acute-care hospitals are scarce, even though these hospitals are often the primary caregiver for children with sepsis. PICU-based studies cannot include these children unless they are transferred to PICUs. Thus, there is an important knowledge gap that must be overcome to better understand real-world clinical practice for community-acquired sepsis in children. We therefore used a national inpatient database in Japan to comprehensively describe mortality, its timing, and risk factors in children admitted to acute-care hospitals with community-acquired septic shock.

## Methods

### Study design and participants

We performed a retrospective cohort study using the Diagnosis Procedure Combination database in Japan. We identified children admitted to acute-care hospitals with community-acquired septic shock from July 2010 to March 2020. Details of the database were described elsewhere [14]; briefly, it includes discharge abstracts and administrative claims data from more than 1200 participating acute care hospitals, which cover approximately eight million inpatient admissions per year, representing more than half of all inpatient admissions to acute care hospitals in Japan. Twenty-four of 27 hospitals with PICUs were registered in the database. The database includes the following data: patient age, sex, body height, weight, Japan Coma Scale score, diagnosis, preadmission comorbidities, post-admission complications, medications, discharge status, and unique identifiers for hospitals. Diagnoses are recorded using the International Classification of Diseases 10th revision (ICD-10) codes. The validity of diagnoses and procedures in the database was established in a previous study; the specificity of the recorded diagnoses exceeded 96%, whereas the sensitivity ranged from 50 to 80%, and the specificity and sensitivity of recorded procedures both exceeded 90% [14]. This study was approved by the Institutional Review Board of the University of Tokyo [approval number: 3501-(5); May 19, 2021] and the procedures were followed in accordance with the ethical standards of the responsible committee on human experimentation and with the Helsinki Declaration of 1975. The requirement for informed consent was waived because of the anonymity of the data.

We chose the following inclusion and exclusion criteria in line with previous research [3, 5, 13, 15]. First, we extracted children younger than 20 years of age who were admitted with sepsis-related diagnoses (ICD-10 codes: A02.1, A28.2, A32.7, A39.4, A40.0–A40.9, A41.0–A41.9, A42.7, A54.8, B00.7, B34.9, B37.7, I30.1, I33.0, J02.0, J20.9, L02.9, L08.0, M86.99, P36.0–P36.9, and T81.4) from July 2010 to March 2020. Among them, we included those with: (i) principal or admission diagnoses including the word ‘sepsis’ or ‘septic’ in Japanese; (ii) antibiotics were started within three days of hospitalization and continued for at least four consecutive days with at least one intravenous dosage; and (iii) vasoactive drugs used within three days of hospitalization. The following exclusion criteria were applied: duration of antibiotics shorter than four days; discharge within three days of hospitalization; transport to another hospital within four days of hospitalization; birth admission; hospitalization on the first day of life; admission with exogenous diagnoses (such as trauma, burn, and toxication); or out-of-hospital cardiac arrest. We did not exclude cases if death was the cause of the short duration of hospital stay and antibiotics use (as in previous studies) [16].

### Outcomes and patient backgrounds

The primary outcome was in-hospital mortality. The other outcomes included the Japan Coma Scale score at discharge, the activity of daily living at discharge, tracheostomy/gastrostomy during admission, and nasogastric tube feeding/home ventilation/physiotherapy at discharge. Discharge with comorbidities included (i) mechanical ventilation, oxygen therapy, tube feeding, or physiotherapy on the day of discharge to home or the day before discharge to home, (ii) worse Japan Coma Scale score at discharge than on admission, (iii) lower level of activity on the modified Rankin Scale or activities of daily living at discharge than on admission, and (iv) tracheostomy or gastrostomy during admission.

Patient background factors included age, sex, principal and admission diagnoses, underlying conditions (preadmission comorbidities, preadmission activity of daily living, requirement of care at home), clinical characteristics (Japan Coma Scale score on admission, transport from other hospitals, cardiac arrest before hospitalization,), tests (blood culture test and other culture tests), and therapies (antibiotics, vasoactive drugs, corticosteroids, intravenous immunoglobulin, blood product, mechanical ventilation, renal replacement therapy, extracorporeal membrane oxygenation, operations, and resuscitation for in-hospital cardiac arrest). Vasoactive drugs included noradrenaline, dopamine, vasopressin, phenylephrine, adrenaline, dobutamine, and milrinone.

Hospital volume was defined as the number of eligible children in each hospital over the study period and was categorized into tertiles (low, middle, and high). According to the Japanese administrative claims system, accredited intensive care units (ICUs) included PICU, neonatal ICU, general ICU, emergency ICU, and intermediate care unit. We categorized impaired consciousness, as assessed by the Japan Coma Scale, into three groups (severe, moderate, and mild) [17]. This category of ‘severe’, ‘moderate’, and ‘mild’ approximately corresponds to 3–8, 9–13, and 14–15, respectively, on the Glasgow Coma Scale [18, 19]. We categorized underlying conditions according to complex chronic conditions for the ICD-10 code [20]. We defined “care-dependence” according to underlying conditions (e.g., limited level of daily activity, need for daily care, home ventilation, home oxygen therapy, tracheostomy, and tube-feeding).

### Statistical analyses

We used a Cox proportional-hazards regression model to analyze the association between study covariates and in-hospital mortality censored on the thirtieth day of hospitalization. The assumption of proportional-hazard was assured based on Schoenfeld residuals and log–log survival plots. We chose the study covariates based on biological and clinical plausibility, and existing literature. The following covariates were included: age, underlying condition (neuromuscular, cardiovascular, respiratory, congenital/genetic, hematological/malignancy/transplant, care-dependence), septic shock as the admission diagnosis, impaired consciousness on admission, transport from other hospitals, admission to accredited ICU within three days of hospitalization, hospital volume, therapies within three days of hospitalization (mechanical ventilation, renal replacement therapy, extracorporeal membrane oxygenation), use of vasoactive drugs on the first day, and use of antibiotics on the first day [2, 5, 10, 21]. All analyses were performed using STATA 17 (StataCorp LLC, College Station, TX, USA).

## Results

### Patient characteristics and therapies

**Fig. 1:**
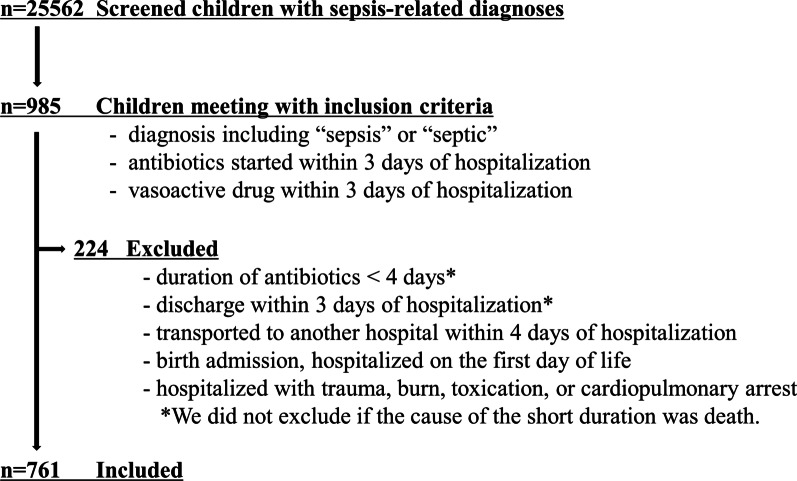
Patient flow

**Fig. 2:**
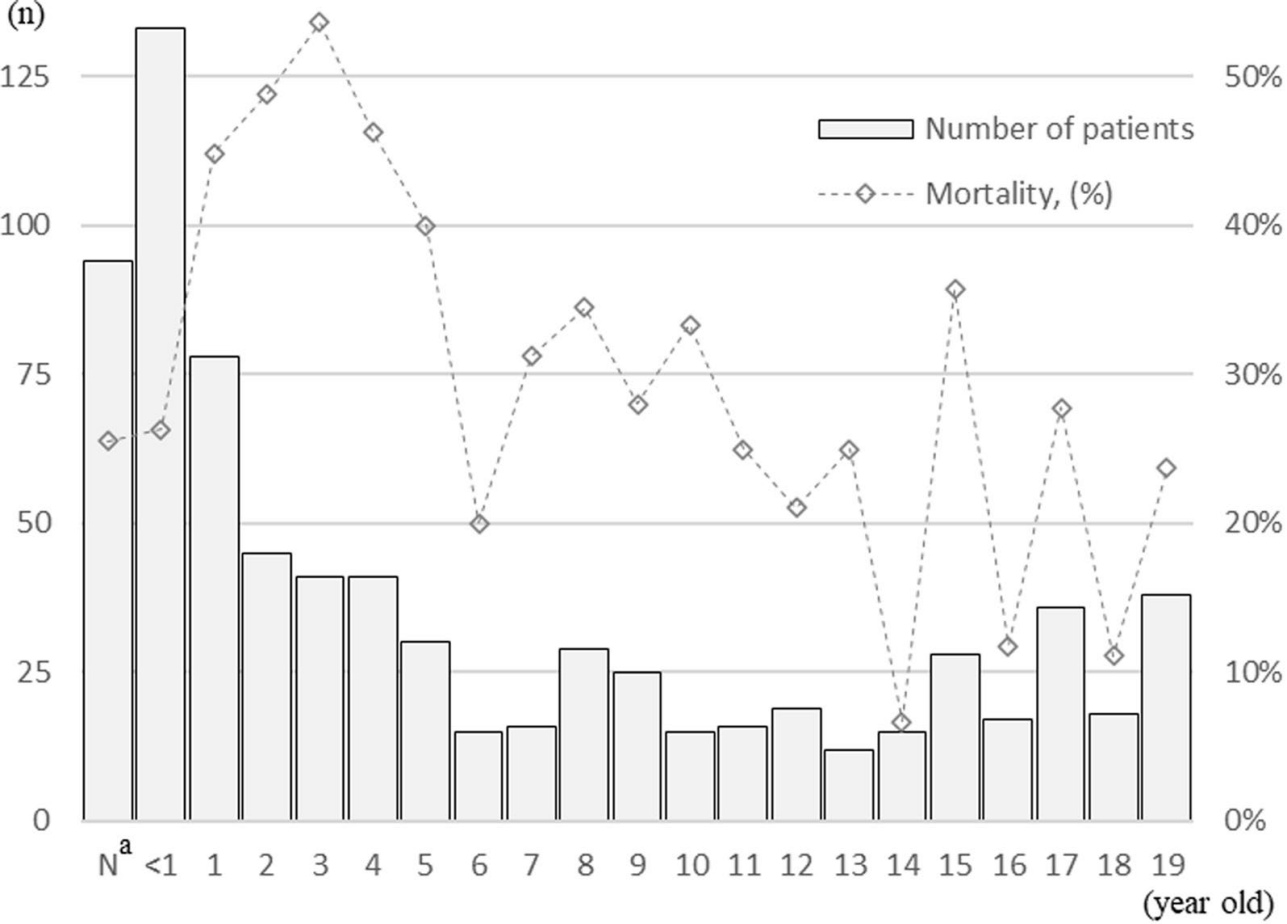
Number of patients and mortality by age (*n* = 761). *N*^a^ indicates neonates of ≤ 28 days of age

We identified 761 children with community-acquired septic shock (Fig. 1). The median age was 3 (interquartile range (IQR), 0 to 11) years. Infants (29 days to < 1 year of age) accounted for 17.4% of the patients (*n* = 133), followed by neonates (12.4%; *n* = 94) and 1-year-old children (10.2%; *n* = 78) (Fig. 2). Among 761 eligible children, 435 (57.2%) children had one or more underlying conditions. Common underlying conditions included care-dependence in 222 (29.2%), neuromuscular conditions in 106 (13.9%) and cardiovascular conditions in 99 (13.0%) children. A total of 511 (67.1%) children were admitted to accredited ICUs within three days of hospitalization and 294 (32.6%) children were transported from other hospitals (Table 1). The 761 children included in this study were treated in 260 hospitals with a median hospital volume of 4 (IQR, 2 to 11).

**Table 1:** Characteristics and outcomes of children with community-acquired septic shock (*n* = 761)

Variables	*n*	(%)
Age		
< 1 year	227	(29.8)
1–5 years	235	(30.9)
6–11 years	116	(15.2)
12–19 years	183	(24.0)
Male	406	(53.4)
Underlying conditions		
Any	435	(57.2)
Care-dependence	222	(29.2)
Neuromuscular	106	(13.9)
Cardiovascular	99	(13.0)
Hematological/malignancy/transplant	58	(7.6)
Congenital/genetic	43	(5.7)
Respiratory	25	(3.3)
Septic shock as the diagnosis at admission	355	(46.6)
Impaired consciousness on admission		
Mild	461	(60.6)
Moderate	52	(6.8)
Severe	248	(32.6)
Transported from other hospitals	294	(38.6)
Admitted to accredited ICU	511	(67.1)
Hospital volume		
1–2	213	(28.0)
3–6	272	(35.7)
7–28	276	(36.3)
Length of mechanical ventilation, days, median (IQR)^a^	7	(3–20)
Length of vasoactive drugs, days, median (IQR)	3	(1–7)
Length of hospital stay, days, median (IQR)	16	(6–39)
In-hospital mortality	244	(32.1)
Discharged with comorbidities^b^	178	(34.4)

Data presented as *n* (%), unless indicated

*IQR* interquartile range

^a^Length of mechanical ventilation in children mechanically ventilated within three days of hospitalization

^b^Proportion of children with new comorbidities among hospital survivors

Mechanical ventilation, renal replacement therapy, and extracorporeal membrane oxygenation were used in 591 (77.7%), 119 (15.6%), and 15 (2.0%) children within three days of hospitalization. Noradrenaline, dopamine, and adrenaline were commonly used vasoactive drugs (Table 2). Blood products, albumin, and corticosteroids were used in 368 (48.4%), 314 (41.3%), and 417 (54.8%) children. Surgical treatment was performed within three days of hospitalization in 26 (3.4%) children. Blood culture tests were performed within three days of hospitalization in in 666 (79.8%) children.

**Table 2:** Therapies within three days of hospitalization (*n* = 761)

	*n*	(%)
Therapies		
Mechanical ventilation	591	(77.7)
Renal replacement therapy	119	(15.6)
ECMO	15	(2.0)
Cardiopulmonary resuscitation	131	(17.2)
Vasoactive drugs		
Noradrenaline	322	(42.3)
Dopamine	380	(49.9)
Vasopressin	92	(12.1)
Adrenaline	307	(40.3)
Dobutamine	231	(30.4)
Milrinone	75	(9.9)
Adjunctive		
Blood products^a^	368	(48.4)
Albumin	314	(41.3)
Corticosteroid	417	(54.8)
Immunoglobulin	290	(38.1)

*ECMO* extracorporeal membrane oxygenation

^a^Blood products include red blood cells, platelets, and fresh frozen plasma

### Mortality and timing

Among 761 children, 244 died (in-hospital mortality rate, 32.1%). Among these 244 cases, 77 (31.6%) children died on the first day of hospitalization, and 156 (63.9%) children died within three days of hospitalization.

### Risk factors for in-hospital mortality

In a Cox proportional-hazard regression model, the risk of earlier mortality increased with a low hospital volume [hazard ratio (HR), 1.94; 95% confidence interval (CI), 1.36 to 2.76; *p* < 0.001], age 1–5 years (HR, 1.90; 95% CI 1.33 to 2.70; *p* < 0.001), severely impaired consciousness (HR, 1.49; 95% CI 1.09 to 2.02; *p* = 0.01), mechanical ventilation within three days of hospitalization (HR, 3.26; 95% CI 2.00 to 5.30; *p* < 0.001), extracorporeal membrane oxygenation within three days of hospitalization (HR, 2.22; 95% CI 1.08 to 4.59; *p* = 0.03), and use of vasoactive drugs on the first day (HR, 1.91; 95% CI 1.37 to 2.68; *p* < 0.001). On the other hand, the risk decreased with admission to accredited intensive care units (HR, 0.52; 95% CI 0.38 to 0.70; *p* < 0.001), transport from other hospitals (HR, 0.71; 95% CI 0.53 to 0.97; *p* = 0.03), care-dependence (HR, 0.57; 95% CI 0.41 to 0.80;* p* < 0.01), and antibiotics started on the first day (HR, 0.36; 95% CI 0.26 to 0.50; *p* < 0.001), as described in Table 3.

**Table 3:** Factors associated with mortality in the Cox proportional hazards analysis

Variables	Hazard ratio (95% CI)	*p*
Age		
< 1 year	Reference	
1–5 years	1.90 (1.33–2.70)	< 0.001
6–19 years	0.92 (0.62–1.36)	0.679
Underlying conditions		
Care-dependence	0.57 (0.41–0.80)	< 0.01
Neuromuscular	1.29 (0.90–1.85)	0.17
Cardiovascular	0.89 (0.58–1.36)	0.59
Hematological/malignancy/transplant	0.67 (0.35–1.28)	0.22
Congenital/genetic	1.09 (0.55–2.7)	0.81
Respiratory	1.04 (0.47–2.28)	0.92
Septic shock as admission diagnosis	0.96 (0.72–1.27)	0.76
Impaired consciousness		
Mild	Reference	
Moderate	0.98 (0.56–1.73)	0.95
Severe	1.49 (1.09–2.02)	0.01
Transported from other hospitals	0.71 (0.53–0.97)	0.03
Admitted to accredited ICU	0.52 (0.38–0.70)	< 0.001
Hospital volume		
1–2	1.94 (1.36–2.76)	< 0.001
3–6	1.26 (0.89–1.79)	0.19
7–28	Reference	
Therapies within three days of hospitalization		
Mechanical ventilation	3.26 (2.00–5.30)	< 0.001
Renal replacement therapy	0.97 (0.64–1.47)	0.90
ECMO	2.22 (1.08–4.59)	0.03
Use on the first day of hospitalization		
Antibiotics	0.36 (0.26–0.50)	< 0.001
Vasoactive drug	1.91 (1.37–2.68)	< 0.001

*CI* confidence interval, *ECMO* extracorporeal membrane oxygenation

### Comorbidities

Of 761 included children, 43 (5.7%) received tracheostomy, with the median timing being the 27th (IQR, 17th to 53rd) day of hospitalization. Ten (1.3%) children received gastrostomy with the median timing being the 55th (IQR, 49th to 72nd) day of hospitalization.

Among 517 hospital survivors, 178 (34.4%) children were discharged with new comorbidities; requirement for respiratory support in 80 (15.5%), tracheostomy in 31 (6.0%), decreased consciousness in 10 (1.9%), tube-feeding in 70 (13.5%) and physiotherapy-dependence in 75 (14.5%) children.

## Discussion

In this analysis of children with community-acquired septic shock in acute-care hospitals, we described in-hospital mortality, early death, and risk factors. The in-hospital mortality rate was 32.1% in this study. Cohort studies from 52 European PICUs and 12 American PICUs in academic hospitals showed in-hospital mortality rates of 10% and 9% in children with community-acquired septic shock [12, 13]. Even in an observational study in nine Japanese PICUs, the in-hospital mortality rate was 18% in 44 children with community-acquired severe sepsis or septic shock [11]. These comparisons could indicate that children with community-acquired septic shock in acute-care hospitals have increased mortality in comparison to those in PICUs. We are not entirely sure of the reasons behind this difference. The difference in mortality may be attributable to multiple factors, such as patient selection, microbiological profiles, and healthcare systems (e.g., types of hospitals caring for children with sepsis and compliance to the guidelines for sepsis) [22, 23]. Our analysis included five times the number of children per year in comparison to the Japanese PICU-based study [11]. Therefore, one potential reason could be that PICU-based studies may have described selected patients given there could be rapidly deteriorating children in acute-care hospitals who did not have a chance to be transported to PICUs, which may have contributed to increased mortality and early death in our study. This hypothesis could be supported by a UK study reporting that 26% of deaths occurred before the PICU admission among children referred to a pediatric inter-hospital transport service with a working diagnosis of sepsis [6]. Moreover, in our study, transport from other hospitals was associated with decreased mortality. This study could suggest that future studies could benefit by including not only children admitted to PICUs but also those in more comprehensive settings, which were acute-care hospitals in this study, to better describe the real-world clinical practice in relation to community-acquired sepsis in children.

On the other hand, our findings regarding the large number of deaths in acute-care hospitals may not be generalizable to other countries or healthcare systems. There is variation among countries in the types of hospitals caring for children with sepsis, thresholds for referring ill children to PICUs, and locally available medical transport services [24, 25]. In addition, microbiological profiles could vary; even the frequency of meningococcal infections in children admitted to acute-care hospitals with community-acquired septic shock has not been well documented, although it has been deemed a common pathogen in children with sepsis who show rapid deterioration [6].

We found that two-thirds of deaths occurred within three days of hospitalization. This was higher than the previously reported rates of early death (30–50%) in children with severe sepsis or septic shock [4–6]. Early death was more common in children with septic shock in a North American study, which may explain the high proportion of early death that we observed [5]. This result could emphasize the importance of initial resuscitation for sepsis again. The success of initial resuscitation in local hospitals could reduce mortality in children with sepsis [22]. Moreover, in this study, more than two-thirds of deaths occurred in hospitals in which the hospital volume was less than one case per year. The early deaths that were sporadically distributed to low-volume hospitals could warrant future efforts to enhance the quality of initial resuscitation in these low-volume hospitals [24].

We found that earlier mortality increased with a low hospital volume and in children of 1–5 years of age after adjusting for patient severity and treatments applied, whereas earlier mortality decreased with admission to an accredited ICU and transport from other hospitals. There is some evidence supporting these findings. A North American study showed decreased mortality in high-volume PICUs [5]. Early admission to ICUs might prevent suboptimal resuscitation [23]. Children with community-acquired septic shock could be better treated in accredited ICUs that are well-staffed and which accommodate a large number of critically-ill children. Thus, it is important—from a public health perspective—to ensure a healthcare system in which these critically ill children can be treated in well-staffed ICUs with a high patient volume.

The present study is associated with several limitations. As physiological and laboratory data were limited in the database, we could not describe end-organ dysfunctions according to the International Pediatric Sepsis Consensus Conference definitions [15]. In addition, we could not describe the pathogen, its susceptibility to antibiotics, the timing of recognition of sepsis, or compliance to the guidelines for sepsis, which should be described in future studies to complement our findings. Second, our method of using the ICD-10 codes for sepsis could lead to a narrower cohort with higher mortality in comparison to other coding methods, such as a method extracting data from all children with infection-related diagnoses [26]. However, we considered that including a pure cohort with the diagnosis of sepsis is the safest practically-available approach because it is hardly possible to extract eligible patients from all inpatients with infection-related diagnoses without detailed data on end-organ dysfunctions. Moreover, this approach meant that our study was in line with previous literature [3]. Third, the mortality in this study may have been overestimated because of our inclusion criteria. That is, we only included patients who used antibiotics for four days or more, and thus we may have excluded non-bacterial infections (i.e., viral infections) or milder cases with septic shock. Also, we only included patients who used vasoactive drugs as the surrogate criterion for cardiovascular dysfunction (as in previous studies), and thus we might have excluded milder cases treated with fluid resuscitation alone [15, 27]. Fourth, as for the inclusion criteria, other studies used blood culture results as an inclusion criterion, while we considered that the diagnosis of sepsis was a more specific sign of suspected or confirmed infections [15, 16]. Lastly, as for study covariates associated with mortality, we could not conclude whether these covariates were triggers of deterioration or simply markers of disease severity.

## Conclusion

The mortality of children admitted to acute-care hospitals due to community-acquired septic shock was as high as 32% and early death was common, even in a high-income country with national health insurance. The risk factors in this study could warrant future efforts to enhance the quality of initial resuscitation for sepsis, especially in low-volume hospitals, which were associated with mortality, and to ensure a healthcare system in which children with community-acquired septic shock can be treated in accredited intensive care units.

## Data Availability

Not applicable.

## Abbreviations

PICU: Pediatric intensive care unit
ICD-10: International Classification of Diseases 10th revision
ICU: Intensive care unit
IQR: Interquartile range
HR: Hazard ratio
CI: Confidence interval
